# Ethnic Differences in Socioeconomic and Health Determinants Related to Self-Rated Health Status: A Study on Community-Dwelling Israeli Jews and Arabs in Old Age

**DOI:** 10.3390/ijerph192013660

**Published:** 2022-10-21

**Authors:** Violetta Rozani

**Affiliations:** Department of Nursing, The Stanley Steyer School of Health Professions, Sackler Faculty of Medicine, Tel Aviv University, Tel Aviv 6997801, Israel; koifmanv@tauex.tau.ac.il

**Keywords:** older adults, ethnicity, self-rated health, socioeconomic status, health-related behaviors, chronic disease

## Abstract

Self-rated health (SRH) is widely used as a proxy for general health status. In old age, SRH has been found to be a strong predictor of morbidity, physical functioning, recovery from illness, use of health services, and mortality. This study was designed to examine differences in socioeconomic and health determinants related to self-rated health status among community-dwelling Jews and Arabs aged 65+ years. Cross-sectional data from 2011 on such Jews and Arabs were extracted from reprehensive National Surveys. The association between socioeconomic and health factors with poor SRH was estimated using three hierarchical logistic regression models. The majority of the respondents were Jews (86%), with a mean age of 73.1 (±6.3) years. The study revealed that older Arabs are disadvantaged according to almost every socioeconomic and health indicator compared to Jews. Poor SRH was significantly associated with age (OR = 1.03, *p* = 0.002), ethnicity (Jews OR = 2.62, *p* < 0.001), unemployment/retirement (OR = 2.03, *p* < 0.001), low income (OR = 3.03, *p* < 0.001), low education (OR = 1.37, *p* = 0.013), absence of physical activity (OR = 2.17, *p* < 0.001), dentures (OR = 1.40, *p* = 0.002), and prevalence of one or more chronic diseases (OR = 4.06, *p* < 0.001). The findings therefore indicated that these factors need to be detected and focused on by health professionals in order to improve the population’s general health status.

## 1. Introduction

Self-rated health (SRH) status has been used worldwide as a measure of subjective health by the World Health Organization since the 1950s and is regularly assessed in national surveys in order to monitor the health status of various populations [1]. Although the factors taken into consideration by SRH are not yet totally understood [2], individual SRH seems to provide a comprehensive perception of health—which can generally be regarded as a complex concept made up of physical, emotional, and other dimensions [2,3]. It can also reflect health behaviors [4,5], psychological and social wellbeing [2], trajectories in health over time [6], socioeconomic conditions, and overall quality of life [3,7]. Among older adults, SRH has been found to be a powerful indicator of general health status [3,8] and a strong predictor of morbidity [9], physical functioning [10], recovery from illness, use of health services [3,11], and mortality [12,13,14]. Moreover, SRH levels are highly correlated to physicians’ assessments of health conditions [15,16].

SRH is assessed by a single question: “In general, how would you rate your health?”, and asks individuals to rate their health on a five-point Likert scale from excellent to poor. Nevertheless, SRH is traditionally analyzed as a binary measure—either “good SRH” (representing “excellent”, “very good” or “good” levels), or “poor SRH” (representing “fair” or “poor” levels)—through logistic regression modeling. Occasionally, it is analyzed by multinomial logistic regression modeling [17] as a three-level variable, with “good SRH” (representing “excellent”, “very good” levels), “moderate SRH” (representing a “good” level), and “poor SRH” (expressing “fair” or “poor” levels). Previous studies using these statistical methods across various communities have reported that a poor SRH is significantly associated with increased age, sex (women), number of chronic conditions (diabetes, cognitive impairment, stroke, heart diseases, etc.), functional status, physical health, ability to cope with existing illnesses, wellbeing, and health behavior factors [1,6,8,9,18,19,20,21,22,23].

Over the last two decades, the differences in SRH levels across ethnic groups have become an intense area of interest for the international scientific community [24,25,26,27,28]—including in Israel [29,30,31,32]. This interest is particularly salient for minorities who not only face unique social and economic challenges but are also more vulnerable to diseases than majority populations [25,30]. Although the health status of the population of Israel is generally good and is better than that of many other OECD countries [33], inequalities in socioeconomic and health indicators persist between the subpopulations of Israeli Arabs and Jews [29,30,31,32,33,34]. Indeed, despite universal coverage and national health insurance law existing in Israel since 1995, the Arab society in Israel is an example of an ethnic group with large health disparities when compared to the general population; this is manifested by a wide array of gaps in health care service [35] and, consequently, as a higher incidence and prevalence of chronic diseases [35,36]. In comparison with Israeli Jews, Israeli Arabs tend to visit family physicians and be hospitalized more often but tend to visit specialist physicians less often [35]. Additionally, Arabs develop heart failure [37] and diabetes at a much younger age compared with their Jewish counterparts and have a higher prevalence of diabetes and obesity [38]. Differences remain between Arabs and Jews for specialist and dental care, choice of health plan, health expenditure, visits to private physicians, and use of pharmaceuticals [35].

Periodic health surveys (every 2–5 years) are conducted in Israel by the Health Surveys Unit of the Israel Center for Disease Control (ICDC). These surveys facilitate the monitoring of the population’s health and health-related behaviors over time. Sampling of the population is carried out using computer software and quality control of all completed questionnaires is conducted by the staff of the Health Surveys Unit. Surveys are based on nationally representative samples of between 6000–10,000 Israelis aged 21 and over and are conducted by a team of trained interviewers in both Hebrew and Arabic [39]. This offers the opportunity to evaluate on a large scale whether the relationship between several factors and poor SRH holds across older adults over 65 years old in these ethnic groups. Therefore, the aim of the study was twofold: (1) To identify differences between Jews and Arabs in old age regarding their socioeconomic and health indicators and to evaluate SRH; and (2) to examine the association between socioeconomic factors, health-related behaviors, and common chronic diseases in old age with SRH in these two populations.

## 2. Materials and Methods

### 2.1. Study Population and Source of Data

Data on 2011 (1013 men and 998 women) community-dwelling Jews and Arabs aged 65 years and above were extracted from consecutive cross-sectional Knowledge, Attitudes, and Practices (KAP) surveys conducted by structured personal telephone interviews in 2004 (*n* = 688), 2006 (*n* = 575) and 2008 (*n* = 748). A series of KAP national surveys with participants aged 18 and above were initiated in 1994 in Israel. The surveys were conducted by structured telephone interviews every two years. Since 1996, these surveys have also been expanded to include the Arab population in Israel, and are conducted in both Hebrew and Arabic [39].

### 2.2. The Dependent Variable

The measure of self-rated health in this study was a single question asked at each interview: “At present, would you say that your health is poor, fair, good, very good, or excellent”? Answers were coded as 1 through 5, respectively. For the purpose of the analyses, this was further categorized into two levels: “good” (by gathering “good”, “very good” or excellent”) and “poor” (by gathering “poor” and “fair”).

### 2.3. The Independent Variables

The independent variables represented three main domains, addressing socioeconomic factors, health-related behaviors, and chronic diseases. The following list of these variables was available in the KAP datasets with no missing values.

1. Socioeconomic variables included monthly income (less than the national average, average, or above average); education (less than elementary school, elementary school, or more than elementary school); employment status (employed or unemployed/retired); marital status (currently married/cohabitating or not currently married/cohabitating); and household (number of rooms).
2. Health-related behaviors variables included performing light or moderate physical activity (i.e., at least 30 min of physical activity for fewer than three times per week or for three or more times per week, respectively); sleeping (less than 6 h or 6 h or more per night); smoking status (current smoker or not currently smoking); oral status (dentures or own teeth); skin examination by a physician in the last two years (yes or no); blood pressure examination in the last year (yes or no); and blood cholesterol examination in the last two years (yes or no). Body mass index (BMI) was derived from self-reported height and weight (weight in kilograms divided by height in meters squared, classified into two categories of <30.0 kg/m^2^ or ≥30.0 kg/m^2^).
3. The prevalence of chronic diseases was derived from self-reported answers (yes/no) to having the following diagnoses: high blood pressure (HBP), cardiac diseases (e.g., congestive heart failure, ischemic heart disease, etc.), diabetes, stroke, malignancy (any type), depression (including anxiety disorders), asthma, and high blood cholesterol levels (hyperlipidemia). For the purpose of analysis, the total number of reported chronic diseases was calculated and then dichotomized into “no chronic diseases” and “one or more chronic diseases”.

### 2.4. The Background Variables

Three background variables were included in the study: age (years), sex (men/women), and ethnicity (Jew/Arab).

### 2.5. Ethics

The Ethics Committee of Tel Aviv University approved the study (No. 0005195-1).

### 2.6. Statistical Analyses

Descriptive statistical analyses were performed to describe the general characteristics of the study population. Continuous variables were summarized as minimum, maximum, mean (standard deviation), median, and interquartile range (IQR); categorical variables were expressed as frequencies with percentages. Differences between Jews and Arabs were tested by chi-square or *t*-test, as appropriate with the research variables. Logistic regression analyses were conducted as follows: firstly, the association between socioeconomic factors, health-related behaviors, and chronic diseases and SRH were modeled separately for each variable (with good SRH as the reference) and adjusted for background variables (age, sex, and ethnicity). All variables, except age, were dichotomized. Only the variables found to be significantly associated with SRH were included in the hierarchical logistic regression modeling. Adjusted odd’s ratios (aORs) with 95% confidence interval (95% CI) were used as a measure of the effects of socioeconomic factors (model 1), health-related behaviors (model 2), and chronic diseases (model 3) on poor SRH. Multicollinearity between independent variables was identified by correlation matrix, tolerance, and Variance Inflation Factor (VIF) values [40]. The correlation matrix illustrated low correlations between explanatory variables (ranging from 0.029–0.378). Tolerances for all the predictors were very close to 1 and all the VIF values were smaller than 2.5. Therefore, it can be concluded that multicollinearity was not a concern in the current study [40]. The level of significance was set at a *p*-value of 0.05. The Statistical Package for the Social Sciences version 27 (SPSS Inc., Chicago, IL, USA) software package of R version 4.0.3 (R Core Team, Vienna, Austria) was used for all data analyses.

## 3. Results

### 3.1. Descriptive Analyses

The majority of respondents were Jews (86%), with a mean age of 73.1 (±6.3) years. The Jews were older than the Arabs (73.3 ± 6.5 years vs. 71.4 ± 6.6 years; *p* < 001). Other significant differences were found between Jews and Arabs. Table 1 summarizes the descriptive statistics and bivariate analysis of the study population. For example, Jews were significantly more educated (t (2009) = −20.832, *p* < 0.001) and had higher incomes (X^2^ = 74.076; *p <* 0.001) than Arabs. Addressing health-related behaviors, Jews did more regular physical activity (X^2^ = 79.313; *p* < 0.001) and reported more examinations of blood pressure (X^2^ = 9.856; *p =* 0.002) and skin (X^2^ = 18.442; *p* < 0.001). Jews had more cancer (X^2^ = 23.191; *p* < 0.001) and depression/anxiety disorders (X^2^ = 3.174; *p* = 0.043) than the Arabs. Lastly, more Arabs than Jews were currently married or cohabitating (X^2^ = 10.464; *p* = 0.001), were current smokers (X^2^ = 7.581; *p* = 0.005), had dentures (X^2^ = 93.721; *p* < 0.001), were obese (X^2^ = 5.760; *p* = 0.012), and had diabetes (X^2^ = 27.712; *p <* 0.001) or asthma (X^2^ = 4.617; *p* = 0.024).

### 3.2. Logistic Regression Analysis

Table 2 presents the effects of the independent variables on SRH in the hierarchical logistic regression modeling. The results show that a poor SRH was significantly associated with advanced age (OR = 1.03; *p* = 0.002), ethnicity (Jews (OR = 3.03; *p* < 0.001)), current unemployment status (OR = 2.07; *p* < 0.001), low income (OR = 1.70; *p* < 0.001), and low education (OR = 1.37; *p* = 0.013). The absence of regular physical activity (OR = 2.17; *p* < 0.001), sleeping less than 6 h per night (OR = 3.03; *p* = 0.021), oral status (dentures (OR = 1.40; *p* = 0.002)), and having at least one of the following chronic conditions: HBP, cardiac diseases, diabetes, stroke, malignancy, depression, asthma, or hyperlipidemia (OR = 4.06; *p* < 0.001), were also significantly associated with poor SRH. Obesity had a borderline significant association with poor SRH in model 2 only (OR = 1.33; *p* = 0.055). Interestingly, there was a null association between family status and sex with poor SRH across all three models.

**Figure 1:**
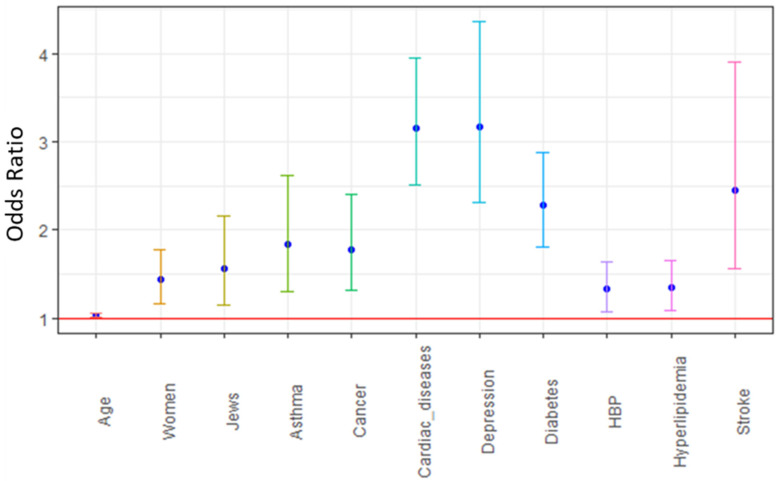
The effect of each chronic disease on self-rated health status among the study population; results from logistic regression modeling (*n* = 2011). Cancer includes any type; Cardiac diseases- include congestive heart failure, myocardial infarct, and ischemic heart disease; HBP—High blood pressure.

Since the prevalence of any chronic disease was significantly associated with poor SRH and as the addition of this single variable resulted in a modest but significant pseudo R^2^ change of 6.5% (Model 3), the last step of the analysis examined the association between each chronic disease, adjusted to control variables (Table 3, Figure 1). This model produced a pseudo R^2^ of 0.253. Moreover, sex (women [OR = 1.44; *p* < 0.001]) was significantly associated with poor SRH in this model only.

## 4. Discussion

At the end of 2008, the population of Israel stood at approximately 7.4 million, of whom 715,300 were older adults aged 65+ years (almost 10% of the total population). Eighty-nine percent of 65+ older adults were Jews and 7.4% were Arabs [34]. This study used a nationally representative large-scale sample of 2011 older Jew and Arab adults in Israel to assess the association between SRH and socioeconomic factors, health-related behaviors, and chronic diseases.

The 1995 National Health Insurance Law aimed to reduce health inequalities among all Israeli citizens by enacting universal health coverage. Every resident is now entitled to a uniform basic basket of services. Yet, Arabs face more obstacles in accessing health care services [35,36], and the findings of the current study particularly reflect the persistence of health inequalities between the Arab and Jewish elderly populations.

Descriptive analyses of the current study revealed that older Arabs were disadvantaged according to almost every socioeconomic and health indicator and are characterized by low income and education and a high level of unemployment. They also suffered from more diabetes and asthma and had more non-healthy behaviors such as smoking and not regularly engaging in physical activity than Jewish Israelis. These results reinforce previous studies that have noted inequalities in socioeconomic status, health-related behaviors, and prevalence of chronic diseases between Jews and Arabs in Israel [29,30,31,32,33]. An explanation of these findings among the Arab population may be related to the negative implications of sociopolitical issues and geographical districts [31,33,36]. However, the differences in SRH among the Jews and Arabs may also be explained by other factors and intermediate variables that are also not included in this study. There may be cultural factors, ways of life, health literacy, history, and social and family structures [14], and—in some cases—sample-related differences. There is also a wide range of other factors in an individual’s life situation that affect how one rates one’s own health. These factors may be different between individuals, between groups in a society, and between ethnic groups [30,33]. They include psychosocial problems/symptoms, emotional status, psychological distress, personality, and lifestyle, which all warrant further evaluation. However, such analyses were not possible to do in an effective way using the data from the KAP surveys. In summary, the nature of this relationship is not clear and probably depends on individual behavior and social influences, as well as broader socio-cultural norms.

The current study also revealed that the majority of older Israeli Arabs rated their subjective health as good, very good, or excellent, as compared to the Israeli Jews. This finding contradicts some previous studies among ethnic minority groups in the world who were more likely to report poor SRH [24,25,26,27,28]. However, this finding is also supported by other studies [29,31,41]. In surveys, Israeli Arabs tend to report relatively high levels of SRH and lower prevalence of most chronic diseases [35], even though their life expectancy is lower and their morbidity and mortality are higher [29,35,41]. However, these previous studies assessed SRH among only the Arab population aged 25–64 years [29] and 30–71 years [41]. Therefore, the current study’s finding serves to extend this observation to a broader range of Israeli society. Nevertheless, Baron-Epel and colleagues [29] found that “SRH in Jews and Arabs does not necessarily have the same meaning in relation to objective measures of health, and caution should be exercised in the use of this measure in different population groups with different cultures”. Notably, Arabs also tend to rate health system responsiveness higher than Jews do, but there are indications that this may be due to lower expectation levels rather than better levels of care in practice [35]. Furthermore, in the time-dependent analysis that considered changes in SRH between two time points in the Jewish and Arab populations, the results showed that Arab Israelis exhibited a weaker deterioration in SRH compared to Jewish Israelis [42]. These findings may warrant further investigation.

Logistic regression analyses of the current study found that an increased age, non-employment status, absence of regular physical activity, low income, elementary education, having dentures, sleeping less than 6 h a night, and having one or more chronic conditions were significantly associated with poor SRH among Israeli older adults. These results are in line with many other studies that have also reported a significant association between poor SRH and age, income [5,6,18,19,20,21,43], unemployment/retired status, and health-related behaviors—specifically sleeping hours, BMI and physical activity [1,6,9,17,22,44,45,46,47,48,49,50]—and chronic conditions [9,21,22,47,48,49,50,51,52,53]. People with chronic conditions can also experience pain and disability that results in poor SRH [47,48,49,50,51,52,53]. The results of the current study indicated that all chronic conditions reported by participants were significantly associated with poor SRH—this is in accordance with other reports that the prevalence of a chronic disease or multi-morbidity is associated with decreased SRH in all age groups [53]. Hence, our findings and those of other studies highlight the important role of chronic conditions in SRH. Accordingly, future longitudinal studies are warranted to clarify the long-term effects of the severity or multimorbidity of chronic conditions on SRH among older adults. Concerning dentures, previous studies have not assessed the link between having dentures and SRH; however, in accordance with previous reports in the literature, a significant association has been observed between poor oral status, dentures, and reduced quality of life among older adults [51,54,55]. Hence, this finding reinforces the benefit of adding the wearing of dentures as a novel direction when investigating SRH among older adults worldwide in the future. In the current study, the null effect of marital status on SRH was observed in all analyses, which can be explained by sample-related differences.

Some limitations can be noted regarding the study: First, due to the cross-sectional design, it is not possible to determine causality for the associations between socioeconomic status, health-related behaviors, and chronic diseases in old age and SRH in either ethnic group. Another limitation relates to sample size; although this study was a national representative study of Jewish and Arab older adults in Israel, a stratified analysis by ethnicity was not possible, as this sample might not be large enough to examine associations between independent variables and SRH for different ethnic and sex groups. Future research based on larger samples can examine associations according to ethnicity and sex groups. More research is also needed into the psychosocial and specific health-related behaviors belonging to these two ethnicities (e.g., diet, health care-seeking behaviors, etc.), which was not possible in this study as the data was limited to the variables collected in the original survey. An additional key variable that was lacking in the current study was health literacy; several studies have found a strong and positive association between health literacy, health–related behaviors, morbidity, and SRH [56,57,58], Nevertheless, no significant difference between the Arab and Jewish populations was observed for health literacy after adjustment for sociodemographic variables [56]. Thus, there may be no ethnic or cultural differences in health literacy between the two groups. Therefore, additional studies are needed to explore the leading theoretical models concerning the complex mechanisms associated with SRH. Another limitation of this study is that although the data were collected using telephone interviews by trained interviewers, all the data was self-reported by the individuals. This may have affected accuracy—specifically that of personal information (e.g., income, weight) and information related to health-related behaviors and chronic diseases. Despite these limitations, this is the first study of its kind to examine the association between socioeconomic factors, health-related behaviors, and chronic diseases and their relationship with SRH in a reprehensive sample of older Jews and Arabs in Israel.

## 5. Conclusions

The study findings show that socioeconomic factors (i.e., education level and employment status), as well as health behaviors (i.e., physical activity and sleeping hours) and chronic diseases (e.g., cardiac diseases and obesity) have a modest, but significant, contribution in determining SRH among older Jews and Arabs in Israel. Consequently, populations who are retired with low income and education, who avoid physical activity, sleep less at night, have poor oral health and any common chronic diseases (including HBP, cardiac diseases, diabetes, stroke, any type of malignancy, asthma, hypertension, and depression) need to be detected, and their treatment emphasized by health professions in order to improve their general health status. Additionally, it is worthwhile investing in improving educational and employment opportunities in early life, which may have long-term positive effects in reducing health inequalities in future cohorts of disadvantaged populations. Finally, early recognition and timely interventions based on stratifying the risks and personal backgrounds of older Jews and Arabs must be taken into consideration. In this way, health behaviors such as physical activity, healthy sleep habits, and good oral hygiene can be improved to effect positive change.

## Figures and Tables

**Table 1:** Socioeconomic factors, health-related behaviors, and health status of the study population (*n* = 2011).

	Jews (*n* = 1727)	Arabs (*n* = 284)	Test
Self-rated Health N (%)			
Poor	124 (8.2)	23 (8.1)	X^2^ = 21.712
Fair	556 (32.2)	62 (21.8)	*p <* 0.001
Good	753 (43.6)	128 (45.1)	
Very Good	197 (11.6)	46 (16.2)	
Excellent	79 (4.6)	25 (8.8)	
SOCIODEMOGRAPHIC CHARACTERISTICS			
Age (years)			
Min–Max	65–99	65–89	t (2009) = −4.809
Mean (SD)	73.3 (6.3)	71.4 (5.6)	*p* = 0.002
Median (IQR)	72.0 (68.0; 78.0)	70.0 (67.0; 75.0)	
Sex N (%)			
Men	843 (48.8)	170 (59.4)	X^2^ = 11.904
Women	884 (51.2)	114 (40.1)	*p* < 0.001
Marital status N (%)			
Married/cohabitating	1120 (64.9)	212 (74.6)	X^2^ = 10.464
Widowed/divorced/never married	607 (35.4)	72 (25.4)	*p* = 0.001
Education (years)			
Min-Max	0–27	0–25	t (2009) = −20.832
Mean (SD)	12.6 (94.4)	6.75 (5.0)	*p* < 0.001
Median (IQR)	12.0 (10.0; 15.0)	6.0 (3.0; 10.0)	
Educational level N (%)			
0–8 years	345 (20.0)	199 (70.1)	
9–12 years	591 (34.2)	35 (18.7)	
13+ years	791 (45.8)	32 (11.3)	
Monthly Income N (%)			
<Average	759 (43.9)	203 (71.5)	X^2^ = 74.076
≥Average	968 (56.1)	81 (28.5)	*p* < 0.001
Current employment status N (%)			
Employed	231 (13.4)	29 (10.2)	X^2^ = 2.170
Unemployed/Retired	1496 (86.6)	225 (89.8)	*p* = 0.082
Household N (%)			
Number of Rooms < 3 rooms	976 (56.5)	160 (56.3)	X^2^ = 0.03
Number of Rooms ≥ 3 rooms	751 (43.5)	127 (43.7)	*p* = 0.503
HEALTH-RELATED BEHAVIORS			
BMI N (%)			
<30 kg/m^2^	1497 (86.7)	231 (81.3)	X^2^ = 5.760
≥30 kg/m^2^	230 (13.3)	53 (18.7)	*p* = 0.012
Smoking Status N (%)			
Current smoker	193 (11.2)	48 (16.9)	X^2^ = 7.581
Non-smoker/former smoker	1534 (88.8)	236 (83.1)	*p* = 0.005
Hours sleep at night N (%)			
<6 h	924 (53.5)	143 (50.4)	X^2^ = 0.972
≥6 h	803 (46.5)	141 (49.6)	*p* = 0.178
Physical Activity Status N (%)			
Active < 3 times per week	708 (41.0)	197 (69.4)	X^2^ = 79.313
Active ≥ 3 times per week	1019 (59.0)	87 (30.6)	*p* < 0.001
Oral status N (%)			
Own teeth	118 (68.4)	110 (38.7)	X^2^ = 93.721
Dentures	545 (31.6)	174 (61.3)	*p* < 0.001
Blood Pressure Examination N (%)			
During last year	1638 (94.8)	256 (90.1)	X^2^ = 9.856
			*p =* 0.002
Blood Cholesterol Examination N (%)			
During last two years	1528 (88.5)	246 (86.6)	X^2^ = 0.809
			*p* = 0.210
Skin Examination N (%)			
During last two years	326 (18.9)	24 (8.5)	X^2^ = 18.442
			*p <* 0.001
CHRONIC DISEASES			
High Blood Pressure N (%)			
No	770 (44.6)	142 (50.0)	X^2^ = 2.884
Yes	957 (55.4)	142 (50.0)	*p* = 0.051
Cardiac Diseases N (%)			
No	1235 (71.5)	212 (74.6)	X^2^ = 1.189
Yes	492 (28.5)	72 (25.4)	*p* = 0.154
High Cholesterol Level N (%)			
No	903 (52.3)	147 (51.7)	X^2^ = 0.032
Yes	824 (47.7)	137 (48.3)	*p* = 0.454
Diabetes N (%)			
No	1354 (78.4)	182 (64.1)	X^2^ = 27.712
Yes	373 (21.6)	102 (35.9)	*p* < 0.001
Stroke N (%)			
No	1632 (94.5)	271 (95.4)	X^2^ = 0.409
Yes	95 (5.5)	13 (4.6)	*p* = 0.318
Cancer (any type) N (%)			
No	1492 (86.4)	274 (96.5)	X^2^ = 23.191
Yes	235 (13.6)	10 (3.5)	*p* < 0.001
Asthma N (%)			
No	1582 (91.6)	249 (87.7)	X^2^ = 4.617
Yes	145 (8.4)	35 (12.3)	*p =* 0.024
Depression/Anxiety disorders N (%)			
No	1511 (87.5)	259 (91.8)	X^2^ = 3.174
Yes	1216 (12.5)	25 (8.8)	*p* = 0.043

**Table 2:** The effect of socioeconomic factors, health-related behaviors, and chronic diseases on self-rated health status among the study population; results from hierarchical logistic regression modeling (*n* = 2011).

Variables	Model 1		Model 2		Model 3	
	Adjusted OR	*p*	Adjusted OR	*p*	Adjusted OR	*p*
	(95% CI)		(95% CI)		(95% CI)	
Age (years)	1.03 (1.02–1.05)	<0.001	1.03 (1.01–1.05)	<0.001	1.03 (1.01–1.05)	0.002
Sex						
Men	-		-		-	
Women	1.09 (0.883–1.34)	<0.001	1.11 (0.89–1.37)	0.352	1.13 (0.91–1.41)	0.272
Ethnicity						
Arabs	-		-		-	
Jews	2.27 (1.66–3.11)	<0.001	3.02 (2.18–4.20)	<0.001	3.03 (2.16–4.26)	<0.001
Current Employment Status						
employed	-		-		-	
unemployed/retired	2.19 (1.56–3.09)	<0.001	2.20 (1.55–3.10)	<0.001	2.07 (1.58–2.95)	<0.001
Marital status						
Married/cohabitating	-		-		-	
Divorced/widows	1.18 (0.948–1.46)	0.14	1.13 (0.90–1.41)	0.288	1.09 (0.87–1.36)	0.482
Monthly Income						
≥Average	-		-		-	
<Average	1.93 (1.58–2.34)	<0.001	1.74 (1.42–2.12)	<0.001	1.70 (1.38–2.09)	<0.001
Education						
More than elementary school (9+ years)	-		-		-	
Less than elementary school (0–8 years)	1.50 (1.19–1.89)	<0.001	1.31 (1.03–1.66)	0.027	1.37 (1.07–1.75)	0.013
Sleep Health						
<6 h			-		-	
≥6 h			1.26 (1.04–1.53)	0.017	1.26 (1.04–1.54)	0.021
Physical Activity Status						
Active			-		-	
Inactive			2.22 (1.90–2.70)	<0.001	2.17 (1.77–2.66)	<0.001
Oral status						
Natural teeth			-		-	
Dentures			1.46 (1.18–1.80)	<0.001	1.40 (1.13–1.75)	0.002
BMI						
<30 kg/m^2^			-		-	
≥30 kg/m^2^			1.31 (0.99–1.72)	0.055	1.18 (0.89–1.56)	0.26
Chronic disease						
No chronic disease					-	
≥1 chronic diseases					4.06 (3.05–5.41)	<0.001
Chi-square of the model	163.092	<0.001	255.592	<0.001	364.324	<0.001
Log likelihood	2524.468		2431.969		2323.324	
Nagelkerke (R^2^)	0.106		0.162		0.225	

**Table 3:** The effect of each chronic disease on self-rated health status among the study population; results from logistic regression modeling (*n* = 2011).

Variable	Univariate			Multivariable		
	OR	95% CI	*p*-Value	OR	95% CI	*p*-Value
Age	1.05	1.03, 1.06	<0.001	1.04	1.02, 1.05	<0.001
Sex			0.002			<0.001
Men	-	-		-	-	
Women	1.33	1.12, 1.60		1.44	1.17, 1.78	
Ethnicity			<0.001			0.004
Non-Jews	-	-		-	-	
Jews	1.59	1.21, 2.09		1.57	1.15, 2.15	
Asthma			<0.001			<0.001
No	-	-		-	-	
Yes	1.90	1.40, 2.59		1.85	1.30, 2.62	
Cancer			<0.001			<0.001
No	-	-		-	-	
Yes	1.93	1.47, 2.52		1.78	1.32, 2.41	
Cardiac diseases			<0.001			<0.001
No	-	-		-	-	
Yes	3.83	3.13, 4.70		3.15	2.51, 3.95	
Depression			<0.001			<0.001
No	-	-		-	-	
Yes	4.23	3.17, 5.68		3.16	2.31, 4.36	
Diabetes			<0.001			<0.001
No	-	-		-	-	
Yes	2.34	1.90, 2.89		2.28	1.80, 2.89	
High Blood Pressure			<0.001			0.007
No	-	-		-	-	
Yes	1.97	1.64, 2.38		1.33	1.08, 1.64	
Hyperlipidemia			<0.001			0.005
No	-	-		-	-	
Yes	1.75	1.46, 2.10		1.35	1.10, 1.66	
Stroke			<0.001			<0.001
No	-	-		-	-	
Yes	3.35	2.24, 5.11		2.45	1.56, 3.91	

## Data Availability

Availability of Data and Materials: The data generated during the current study are available in the Israeli Center for Disease Control ICDC repository, (https://www.health.gov.il/UnitsOffice/ICDC/Health_Surveys/Pages/KAP.aspx (accessed on 26 February 2019)), and from the corresponding author upon reasonable request.
